# The COVID-19 pandemic and Community Health Workers: An opportunity to maintain delivery of care and education for families of children with epilepsy in Zambia

**DOI:** 10.7189/jogh.10.020329

**Published:** 2020-12

**Authors:** Lauren Sham, Ornella Ciccone, Archana A Patel

**Affiliations:** 1 Department of Neurology, Division of Epilepsy & Clinical Neurophysiology. Boston Children's Hospital, Harvard Medical School. Boston, Massachusetts, USA; 2 University Teaching Hospital – Lusaka Children’s Hospital. Lusaka, Zambia; 3 Department of Paediatrics and Child Health, University of Zambia School of Medicine, Lusaka, Zambia

The COVID-19 pandemic has had a significant impact on the delivery of care for chronic neurological diseases globally. As requirements for physical distancing have led to restrictions on the availability of health care services, many countries have adapted methods of telemedicine to sustain care access for patients [1], while making difficult decisions surrounding which aspects of direct clinical care can be deferred and the time span acceptable for delaying chronic medical care [2]. For people with epilepsy, issues such as determining criteria for what constitutes urgent management, managing the risk of increased seizures in the setting of illness, as well as ensuring a stable medication supply, have all been raised as critical concerns during this pandemic [3].

In resource limited settings, there is a paucity of neurologic care at baseline due to limited availability of specialists, diagnostics, and treatment options [4]. During infectious disease outbreaks, such issues become compounded as resources become even more constrained, and alternate care delivery through telemedicine is a limited option at the primary care level in the developing world. In Africa, the vast majority of people are low-income and live in crowded homes where physical distancing is impossible, and transport to obtain essential medications may require use of crowded public transportation. In such situations, risk of disease transmission while accessing health care during a pandemic is of extremely high concern [5]. As a result, continued adherence to anti-seizure medications can become difficult for people with epilepsy, compounding the strain on the system by resulting in potential exacerbation of seizures [6].

However, the workforce in Africa does provide a unique opportunity for care delivery through community health workers (CHWs). CHWs have been a longstanding method of mobilizing people from within a community with varying knowledge bases to deliver basic medical screening and education to those with limited access to clinical care, working through challenges due to differing belief systems, limited knowledge base, geographic barriers, and stigma. Through this system, there has been proven success in improving health seeking behaviors in the community, particularly in conditions such as HIV and epilepsy [7]. In 2019, we launched a CHW program for pediatric epilepsy in the Linda township of Zambia, a region approximately 16 km south of the capital city of Lusaka. Linda compound is a very low income community, comprising of a population of approximately 25 000 people living in fewer than 4000 households total, with only 2000 people holding formal employment. Our program was created in partnership with Neri/i4life clinic, which had an existing CHW program for pediatric nutrition.

The initial goals of our epilepsy CHW program were to improve adherence to treatment for children with epilepsy and provide education to reduce associated stigma and improve overall inclusion in society. We combined a training program for local health providers on epilepsy clinical management with the CHW program, and 10 CHWs were provided training on the basics of seizures, medication adherence, seizure safety in the home, and psychosocial issues, and were prepared for their role as a connection to the clinic for these families of children with epilepsy. The CHWs were then connected with 40 families, whom they visited twice monthly. They were 4 months into the program when the COVID-19 pandemic led to restricted activities in Zambia.

In early March 2020, warnings of COVID-19 on ongoing clinical care were emerging quickly across Africa. During this time, a monitoring site visit of our CHW program was in process. As Zambia reported the first positive cases during that time and initial restrictions for COVID-19 prevention were implemented by the government, it became clear that adaptation of all services would be required. While necessary for safety, our team struggled like many others in this time with the potential impact on care and risk to our patients’ neurologic health. With this in mind, we re-evaluated how the existing infrastructure created from this program could be used in this time.

Neri clinic, like many rural health centers in the region, is a small building with crowded spaces where people wait in close proximity to see clinicians and collect their medications on a monthly basis. Even if solely present for the purposes of medication collection, families would be at high risk for exposure. As adherence to medication is essential for seizure management, we wanted to ensure that families did not have gaps in coverage during this time period. We quickly realized that our CHW program provided an excellent opportunity to deploy individual workers to households for medication distribution, mitigating the risk of families congregating in the clinic.

**Figure:**
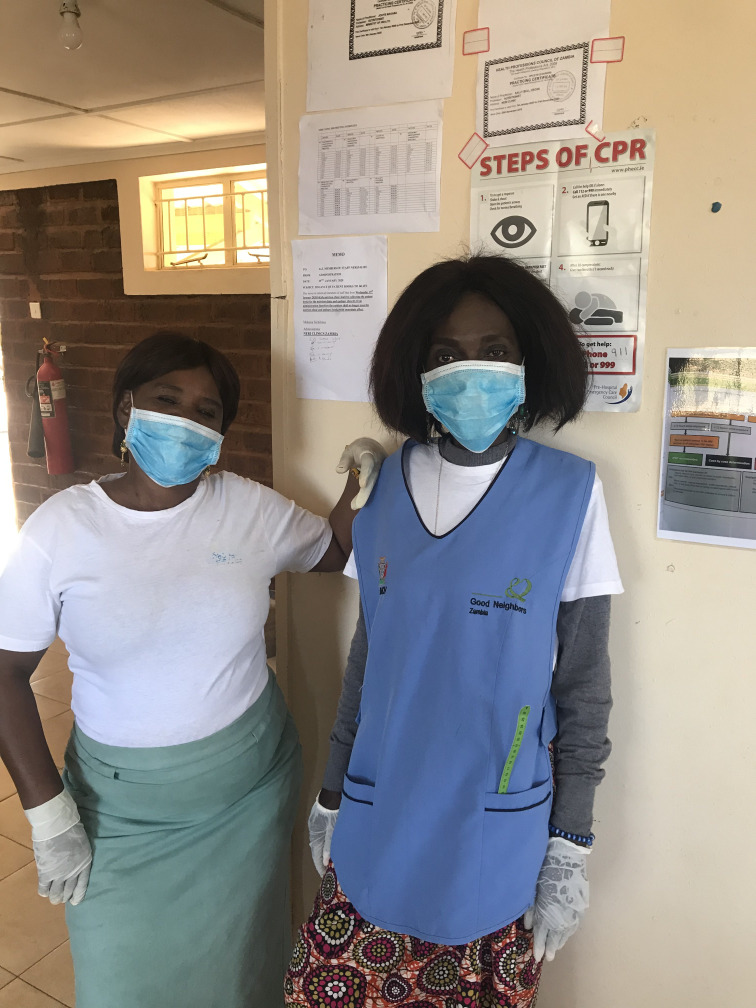
Photo: Photo provided by Sally Bell-Cross of Neri Clinic/i4life clinic (used with permission).

Personal protective equipment and hand sanitizer were provided for each CHW to decrease their own exposure risk. In addition, an educational component surrounding COVID-19 could be delivered to each household by the CHWs. While the Zambian Ministry of Health had provided guidance on appropriate hygiene and social distancing measures, many of our clients are illiterate and have limited access to information. The CHWs were able to deliver up to date education on COVID-19 and social distancing information to families in a reliable manner and in the local dialect with opportunity for discussion. Adaptations to make this process safer included reducing visits from twice monthly to once monthly, and incorporating basic screening questions (with history obtained by CHWs, then reviewed by providers at the clinic) to determine if an urgent clinic visit was necessary, all in efforts to limit exposure but not compromise care for the child’s epilepsy. Antiseizure medication supply gaps, which have been exacerbated by resource shifting and increased purchasing restrictions, have been supplemented by the NGOs supporting the program.

This CHW program has provided a unique opportunity to support the Linda compound community in Zambia during the COVID-19 pandemic. It has been possible to implement a system in which access to essential medications could continue to be provided in a safer manner, and children who were in need of urgent clinician visits could be specifically identified, to reduce overall outpatient volumes and allow spacing of visits for physical distancing. Additionally, we could continue to provide education and support to our families, in alignment with our original objectives for the program. During this time of crisis, we have seen the CHWs in our program continue to be motivated to help their community and continue to see the positive impact our program has on the participating families and the Linda community as a whole. Beyond our original goals, this program has provided a unique model of health care access for a chronic medical condition during times of infectious disease outbreaks requiring physical distancing, which is feasible to implement in a developing region where telehealth mechanisms are limited.
